# Family refusal rates for organ donation after brain death and after circulatory death: a single-center 6-year experience

**DOI:** 10.1186/s44158-025-00301-7

**Published:** 2025-11-21

**Authors:** Federica De Min, Maria Assunta Donato, Elisa Raimondo, Alessandro Ivan De Martino, Alessia Bresil, Alessandra Agnese Grossi, Martina Baiardo Redaelli, Paolo Severgnini, Luca Cabrini

**Affiliations:** 1 General and Neurosurgical Intensive Care Units, Circolo Hospital, ASST Sette Laghi, Varese, Italy; 2 Organ and Tissue Procurement Coordination, ASST Sette Laghi, Varese, Italy; 3 Department of Human Sciences, Innovation and Territory, University of Insubria, Varese-Como, Italy; 4 Center for Clinical Ethics, Department of Biotechnologies and Life Sciences, University of Insubria, Varese-Como, Italy; 5 Department of Biotechnologies and Life Sciences, University of Insubria, Varese-Como, Italy; 6 Cardiological Anesthesia and Intensive Care Unit, Circolo Hospital, ASST Sette Laghi, Varese, Italy

**Keywords:** Transplantation, Organ donation, Intensive care, Ethics

## Abstract

**Background:**

A wide gap exists between the number of patients awaiting organ transplantation and the availability of donor organs. For most vital organs, no artificial substitutes are available, resulting in the death of approximately 3–4% of patients on transplant waiting lists.

Deceased organ donation occurs through one of two pathways: donation after brain death (DBD) or after controlled circulatory death (cDCD). In the absence of documented consent during the patient’s lifetime, many countries rely on family members to make the decision, placing an emotional burden on them during a time of acute grief.

Data on family attitudes toward organ donation, particularly in relation to the mode of death (DBD vs. cDCD), remains limited. This study aimed to examine the frequency of family refusal in the context of deceased organ donation and assess whether it differs between DBD and cDCD cases.

**Methods:**

This retrospective, single-center observational study analyzed 6 years of hospital data (2019–2024) from five intensive care units at a tertiary university hospital in Varese, Italy. All patients, regardless of age, who were eligible for deceased organ donation through either DBD or cDCD were included.

**Results:**

A total of 158 patients were evaluated for donation (135 DBD; 23 cDCD).

Documented opt-in consent, as recorded in the national donor registry, was available for 41 patients (20%), while 3 had registered their refusal. Two patients were ineligible for donation; for the remaining 112 patients (71%), consent was sought from family members. Family refusal occurred in 28 cases (25% of the 112 families asked for consent). No significant difference in consent rates was observed between DBD and cDCD pathways (DBD: 73/98 vs. cDCD: 11/14; *p* = 0.74).

**Conclusions:**

Family refusal was the leading reason for missed donation opportunities among patients without documented opt-in consent, accounting for one in four cases. The donation pathway (DBD vs. cDCD) did not significantly influence family decision-making. Given ongoing organ shortages and high wait-list mortality, strategies are needed to support family decisions and to promote the formal registration of opt-in consent among citizens.

## Background

A substantial global gap exists between the number of patients awaiting organ transplantation and the availability of donor organs, leading to a significant number of preventable deaths every day [1]. At the end of 2019, over 58,000 patients were on transplant waiting lists within the European Union alone [2]. While artificial organ support is widely available for end-stage kidney disease, transplantation remains the only life-saving option for most vital organs, including heart, lung and liver. The persistent shortage of donor organs contributes to an annual mortality rate of 3–4%among patients on waiting lists, with an estimated 10 deaths occurring each day [3].

Several barriers hinder the expansion of organ donation, among which personal and family attitudes play a crucial [4, 5]. In many countries, in the absence of a patient’s registered opt-in/opt-out consent, family members are approached for organ donation authorization. In Italy, this responsibility falls primarily on the spouse and/or children, often imposing a heavy emotional burden at a time of acute grief.

Limited evidence exists on the rate of family refusal when approached for organ donation, particularly when comparing the two primary donation pathways: donation after brain death (DBD) and controlled donation after circulatory death (cDCD) [1, 5–9]. To fill this gap, we evaluated the 6-year experience at a single academic hospital in Italy.

## Methods

### Study design and setting

We performed a retrospective analysis of all cases evaluated for potential organ donation between 2019 and 2024 at the Tertiary University Hospital “Ospedale di Circolo e Fondazione Macchi” in Varese, Italy.

The hospital comprises 5 intensive care units (ICU) (general, mixed, neurocritical, and cardiac), with a total of 36 beds and approximately 2010 annual admissions. About half of the admissions are postoperative patients, a group typically associated with lower mortality, resulting in an overall ICU mortality rate of 9.4%. The first cDCD procedure at our hospital was performed in 2017.

In Italy, organ donation is regulated by national legislation that implements a mixed “opt-in/opt-out” system, which applies equally to DCD and DBD. Citizens may formally register their consent or refusal to donate their organs after death, and their choice is recorded in the national donor registry. As of 31 December 2024, approximately half of the Italian population (58.1%) had expressed a decision at the time of ID renewal, with 70% opting in and 30% opting out [10]. In cases where the deceased has not formally opted out, bereaved family members—typically the spouse or adult children—are approached by healthcare professionals (HCP) in emergency departments or ICUs to obtain organ donation authorization. Legally, a family cannot override the decision of a deceased individual who registered their consent; however, in rare cases of conflict, transplant centers evaluate the situation to identify a respectful and non-confrontational resolution.

### Study population

All patients assessed for organ donation by the Organ Procurement Hospital Organization during the study period were included, regardless of age.

### Data sources and ethics

Patients’ opt-in vs. opt-out consent status was determined by consulting the national donor registry. This retrospective study used patient data (collected for administrative purposes as required by the regional and national agencies regulating organ donation) that had already been anonymized and de-identified in the Organ Procurement Hospital Organization database prior to extraction for analysis. No identifying information about patients or their relatives was accessible to the investigators. Therefore, in accordance with Italian legal regulations (D.L. 196/2003, art. 110; 24 July 2008, art. 13), Ethics Committee approval was not required. The study was conducted in compliance with the principles of the Declaration of Helsinki (with amendments) and the current General Data Protection Regulation (GDPR).

### Statistical analyses

Descriptive statistics were used to summarize the characteristics of the study population. Normality of continuous variables was assessed using the Shapiro–Wilk test. These data are presented as mean ± standard deviation (SD) for normally distributed variables and as median with interquartile range (IQR) for non-normally distributed ones. Continuous variables were compared using the Mann–Whitney *U* test (Wilcoxon rank-sum test) if the assumption of normality was not met. Categorical variables are reported as absolute frequencies and percentages. For categorical variables, comparisons were performed using the chi-square test or Fisher’s exact test, as appropriate. All statistical tests were two-sided, and a *p*-value < 0.05 was considered statistically significant. Statistical analyses were conducted using R Software (R Foundation for Statistical Computing, Vienna, Austria).

## Results

During the study period, 158 patients were evaluated for potential organ donation, including 23 cDCD and 135 DBD. Patient demographics and clinical characteristics, including the primary diagnosis leading to hospital admission, are reported in Table 1. Almost all patients were adults (≥ 18 years old), but 4 were below 18 years old. One was an infant and was admitted and treated at the pediatric intensive care unit, while three other patients were teens and treated in the neurocritical and general ICUs.

**Table 1:** Characteristics of patients considered for donation

	cDCD (*n* = 23)	DBD (*n *= 135)	*p* value
Age, median (IQR)	62(48–70)	59 (47–71)	0.86
Gender, male *n *(%)	17 (74%)	74 (55%)	0.11
Time to death, days median (IQR)	10 (8–12)	3 (2–4)	< 0.001
Not suitable for donation	1 (4%)	9 (6.66%)	1
Primary event	*N*(%)	*N*(%)	
Cardiac arrest	9 (39.1%)	15 (11.1%)	< 0.001
Stroke	7 (30.4%)	80 (59.3%)	0.010
Trauma	4 (17.4%)	25 (18.5%)	1
Other	3 (13%)	15 (11.1%)	0.73

*Abbreviations*: *cDCD *controlled donation after circulatory death, *DBD *donation after brain death

Among the 158 patients assessed for organ donation, patients’ consent was available from the national donor registry in 44 cases (27.8%). In the remaining 114 cases (72.2%), consent was sought from the patients’ family.

Of the 23 patients considered for cDCD, 9 had formally registered opt-in consent, and none had opted out. Among the remaining 14 patients, family consent was obtained in 11, while 3 families opposed donation (21.4% of cases requiring family consent). An additional 3 cDCD procedures could not be performed due to technical limitations. Ultimately, 17 cDCD donations were performed.

During the same period, 135 patients were diagnosed with brain death. Of these, 3 had formally registered opt-out consent and 32 had opted in. Among the remaining 100 patients, 2 were deemed ineligible, and families were not approached for consent. Of the remaining 98 patients, family consent was obtained in 73 cases (54.1%), while 25 families refused (25.5%). An additional 10 cases (10.2%) did not proceed to donation due to medical contraindications or technical reasons (1 case). In total, 95 DBD procedures were performed.

Across all donation types, family consent was granted in 84 of 112 cases where a decision was required (75%). The incidence of family refusal among patients who had not formally registered their opt-in/opt-out consent was similar between the cDCD and DBD groups (Table 2).

**Table 2:** Patient and family consent and refusal to organ donations

	cDCD (*n* = 23) *N*(%)	DBD (*n* = 135) *N*(%)	*p *value
Patient’s consent	9 (39.1%)	32 (23.7%)	0.13
Family consent	11 (47.8%)	73 (54.1%)	0.58
Patient’s refusal	0 (0%)	3 (2.2%)	1
Family refusal	3 (13%)	25 (18.5%)	0.77
	cDCD patients for whom families were asked for consent (*n* = 14)	DBD patients for whom families were asked for consent (*n* = 98)	
Family consent	11 (78.6%)	73 (74.5%)	0.74

*Abbreviations*: *cDCD *controlled donation after circulatory death, *DBD *donation after brain death, *2 DBD *patients were not asked for consent due to ineligibility

Overall, during the study period, 112 donations were performed, representing 71% of all cases considered. Among the 48 patients for whom donation did not proceed, family refusal was the most frequent reason (28 cases; 60.8%), followed by medical ineligibility or technical reasons. Time between hospitalization and death was significantly lower in the DBD group.

## Discussion

To the best of our knowledge, this is the first study in Italy to investigate the incidence of family refusal and consent for organ donation in potential donors following DBD and cDCD. In the absence of prior formal registration of the patient’s wishes about organ donation, families were asked to decide in the majority of cases. Among these, family refusal occurred in approximately one-quarter of cases, making it the most common reason for donation not proceeding. We observed no difference in the incidence of family refusal between DBD and cDCD. Notably, in the vast majority of cases, family consent was required due to the low prevalence of formally registered opt-in/opt-out patient consent.

The predominance of family refusal as a key barrier to organ donation aligns with findings from other European countries. A recent multicenter study in Germany similarly identified family decision-making as a critical determinant of donation outcomes [11]. Understanding the underlying reasons for family refusal is therefore crucial to inform effective interventions aimed at increasing consent rates. Although specific data from Italy remain limited, prior research has suggested that negative emotions, spiritual concerns, uncertainty about brain death, and mistrust in the healthcare system can influence family decisions [12]. Although studies from Europe are scarce, some scholars have identified several factors associated with higher family refusal rates. These include ethnicity (with whites more favorable than Asians or Africans), religious beliefs (with atheists and Christians more favorable than Hindus and Muslims), socioeconomic status (wealthier more favorable), and the sex and age of potential donors (with lower consent rates for female donors and higher consent for pediatric patients). Additional contributing factors include the absence of a specialized organ-donation nurse, and, most notably, a lack of awareness regarding the patient’s wishes about donation [5, 9]. Negative emotions (e.g., “feeling overwhelmed”) and inadequate support from other relatives or healthcare professionals have also been [11, 13–15].

Ethnicity has also been identified as a significant factor influencing attitudes toward organ donation in the United States. Studies have shown that African American and Asian American individuals often require culturally tailored approaches, particularly due to strong family influence and cultural concerns about body integrity, especially among Asian populations [16–18]. Similarly, higher refusal rates have been reported among immigrant populations in Italy, with particularly elevated rates among individuals of Asian origin [19]. The widespread presence of negative attitudes toward organ donation among ethnic and religious minorities may, in part, stem from feelings of social marginalization [4]. Religious objections related to body integrity, intra-family disagreements, fear of social judgment, and mistrust in the healthcare system have also been documented [20, 21]. Despite different attitudes among patients and families toward organ donation from cDCD or DBD, these differences are nuanced and context specific, and data show similar authorization rates. Families could not perceive a major distinction between the two modalities during the decision-making process. On the one hand, the concept of brain death could be more ambiguous and less accepted by some families and cultural groups [22, 23], while on the other hand, cardiac death could be perceived as a clearer indicator of death, which might increase certainty and comfort for some families when considering organ donation [24]. Moreover, deep-rooted concerns about cDCD exist, and this might affect decision making in both healthcare personnel and the general population [25], as well as global inconsistencies in the criteria for declaring brain death continue to contribute to uncertainty among the public and some HCPs [4, 26].

Despite these challenges, there is evidence that consenting to organ donation can have a positive and lasting impact on the grieving process of families. In a Dutch study, regret after consenting was reported by only 10% of families, while refusal led to persistent emotional distress in a larger proportion [27]. Similarly, a previous study from our center indicated that donation often contributes positively to family coping 1 year after the event [19].

A second key—and unexpected—finding of this study is the comparable incidence of family refusals between DBD and cDCD. While this is consistent with some reports, such as a US study that found no significant difference in consent rates between donation types [6], most studies reported greater support for DBD [5, 9, 16, 28, 29]. Some investigations have reported lower refusal rates for both cDCD and uncontrolled DCD relative to DBD [7, 30], while others observed higher consent rates for uncontrolled DCD compared to cDCD [8].

The time between hospitalization and death differed between patients undergoing cDCD and DBD, with those in the controlled cardiac death group experiencing a longer course. This difference is likely related to the clinical trajectory: catastrophic brain injury or hypoxic–ischemic insult typically progresses to brain death within a few days, whereas in controlled cardiac death, organ donation follows a planned withdrawal of life-sustaining therapies, which requires prior prognostic assessment and sufficient time for evaluation. Our study highlights the fundamental impact of family refusal on donation rates. A substantial increase in donation rates could be achieved through strategies aimed at reducing family refusal. These may include public awareness campaigns to clarify the concept of brain death, promote trust in healthcare institutions, and normalize the act of donation. Tailoring these efforts to cultural and religious sensitivities is essential. Furthermore, encouraging individuals to express and share their donation preferences during their lifetime may relieve families of the emotional burden of making decisions in crisis situations.

### Limitations and strengths

This study has some limitations. It was a retrospective, single-center analysis based on administrative records, which did not include information on the specific reasons for family refusal, nor on potential influencing factors such as ethnicity or religious beliefs. Future research should investigate the reasons underlying family refusal using qualitative approaches (e.g., interviews or focus groups) and/or quantitative methods (e.g., surveys). Multicenter, prospective studies are also needed to enhance the generalizability of the findings. In addition, greater attention should be given to cultural and religious influences on decision-making, with the aim of developing communication strategies tailored to the needs of specific groups [31]. The relatively small number of cases certainly limited the possibility of detecting significant between-group differences. Finally, the effectiveness of public awareness campaigns and family support initiatives should be systematically assessed. Despite these limitations, our study represents the first attempt in Italy to quantify and compare family refusal rates across both DBD and cDCD over a substantial 6-year observation period. Notably, the predominance of DBD cases reflects the relatively recent implementation of DCD protocols in Italy, following national approval in 2008. Consistent with national data, DBD remains the predominant donation pathway in Italy [10].

## Conclusions

In this 6-year retrospective study, family consent was sought in the majority of organ donation cases, and family refusal occurred in approximately a quarter of these. Family refusal was the predominant reason for the failure to proceed with donation, and the incidence did not differ between DBD and cDCD cases. These findings underscore the critical importance of addressing family attitudes, knowledge, and emotional readiness through targeted public health interventions and culturally sensitive communication strategies. Promoting the explicit declaration of donor preferences during life may further reduce the burden on families and enhance overall donation rates.

## Data Availability

Data will be available upon reasonable request to the corresponding author.
